# A System of Human Anatomy, General and Special

**Published:** 1844-10

**Authors:** 


*A System of Human Anatomy, General and Special*, by Erasmus Wilson, Lecturer on Anatomy, London. Second American edition. Edited by Paul B. Goddard, M. D., &c. With 200 illustrations by Gilbert. Philadelphia, Lea & Blanchard, 1844. (From the Publishers.)

Having already recommended the above work to students as a text book, to the course on Anatomy in the Rush Medical College, nothing we can here say will express a higher opinion of its merits. Nothing of the size could be more perfect. D. B.

